# *Duchesnea indica* Extract Ameliorates LPS-Induced Septic Shock in Mice

**DOI:** 10.1155/2022/5783867

**Published:** 2022-05-14

**Authors:** Yuan Yee Lee, Heung Joo Yuk, Evelyn Saba, Sung Dae Kim, Dong-Seon Kim, Spandana Rajendra Kopalli, Jae-Wook Oh, Man Hee Rhee

**Affiliations:** ^1^College of Veterinary Medicine, Kyungpook National University, Daegu 41566, Republic of Korea; ^2^Herbal Medicine Research Division, Korea Institute of Oriental Medicine, Daejeon 34054, Republic of Korea; ^3^Department of Veterinary Biomedical Sciences, Faculty of Veterinary and Animal Sciences, Pir Mehr Ali Shah Arid Agriculture University, Rawalpindi 46000, Pakistan; ^4^Department of Integrative Bioscience and Biotechnology, Sejong University, Gwangjin-Gu, Seoul 05006, Republic of Korea; ^5^Department of Stem Cell and Regenerative Biotechnology, KIT, Konkuk University, Seoul 05029, Republic of Korea

## Abstract

**Objective:**

*Duchesnea indica* has been reported for its anti-inflammatory properties. However, its efficacy in sepsis has yet to be reported. In this study, we studied the ability of *Duchesnea indica* extract (DIE) to rescue mice from septic shock and sepsis.

**Methods:**

*In vitro* studies included the measurement of secreted nitric oxide, cell viability, gene and protein expression via real-time polymerase chain reaction and western blot, and confocal microscopy in RAW 264.7 cells. *In vivo* studies include a model of septic shock and sepsis in BALB/c mice induced by a lethal and sub-lethal dose of lipopolysaccharide (LPS).

**Results:**

DIE suppressed the expression of proinflammatory cytokines induced by LPS and prevented the translocation of NF*κ*B into the nucleus of RAW 264.7 cells. It also prevented reactive oxygen species damage induced by LPS in murine bone marrow-derived macrophages. Models of sepsis and septic shock were established in BALB/*c* mice and DIE-rescued mice from septic shock. DIE also reversed the increase in tumor necrosis factor-*α* and nitrite levels in the serum of mice induced with sepsis. DIE also prevented the translocation of NF*κ*B from the cytosol into the nucleus in murine lungs. Histopathological damage induced by sepsis was reversed in the testis, liver, and lungs of mice.

**Conclusion:**

In conclusion, DIE is a suitable candidate for development as a therapeutic agent for sepsis.

## 1. Introduction

In the Third International Consensus definition for Sepsis and Septic Shock (Sepsis-3) in 2016, sepsis is defined as a life-threatening disease that causes organ dysfunction because of a dysregulated host response to infection. Septic shock has been defined as a subset of sepsis in which underlying circulatory, metabolic, and cellular abnormalities are sufficient to cause death [1]. Antibiotics like ceftriaxone and metronidazole were prescribed in combination to patients with sepsis [2, 3], but a study has shown that ceftriaxone induces an altered gut microbiota in mice [4]. Side effects of consuming pharmaceutical drug is unavoidable. Natural drugs contain active ingredients like polysaccharides, organic acids, flavonoids, and saponins that have been widely reported to benefit and suppress various diseases [5].

Pathogen-associated molecular patterns (PAMPs) and damage-associated molecular patterns (DAMPs) are detected by receptors such as the toll-like receptors (TLRs). TLRs are known to activate the nuclear factor *κ*B (NF*κ*B) and the mitogen-activated protein kinase (MAPK) pathway [6]. LPS is a component of the outer membrane of Gram-negative bacteria and is a ligand for TLR4 [7]. Hence, LPS and TLR4 signaling are suitable targets for elucidating the efficacy of herbal extracts against sepsis. NF*κ*B is also known to be associated with various inflammatory diseases [8, 9]. Therefore, we targeted NF*κ*B as a primary determinant in this study.

*Duchesnea indica*, commonly known as false strawberry, is normally found in east Asia, in particular, China and Japan. It has been reported that the leaves and whole plant exhibit anticancer [10, 11], antioxidant [12], and immune modulatory activities [13]. There have been no studies specifically evaluating the efficacy of *Duchesnea indica* extract (DIE) as a therapeutic agent for sepsis. However, the whole plant extract has been reported to exhibit anti-inflammatory activities in RAW 264.7 cells [14]. Zhu et al. (2014) have identified a total of 27 phenolic compounds from *Duchesnea indica* ranging from ellagic acid glycosides, ellagitannins, ellagic acid, hydroxybenzoic acid, flavonoids, and hydroxycinnamic acid derivatives [15]. In another study, compounds like kaempferol, apigenin, and arjunic acid were also identified in the ethyl acetate fraction of ethanol extracts of *Duchesnea indica* [16]. Flavanoids and kaempferol were also well-known for their antioxidant activity [17, 18], indicating the potential benefits of DIE. This shows that DIE may contain a variety of bioactive compounds that are therapeutic against various diseases.

As sepsis represents a dysregulation of the host response to infection and a flared inflammatory response, we hypothesize that DIE may be an effective treatment for sepsis. Reactive oxygen species (ROS) are produced by cells that are involved in the host defense response [19] and are also closely associated with hypoxia and cancer [20]. As DIE was reported for its potent antioxidant properties, and sepsis is also associated with the imbalance of oxidants and antioxidants [21], it may indicate that DIE is capable of attenuating sepsis. Our study shows that DIE has the potential to be developed into a therapeutic agent for sepsis.

## 2. Materials and Methods

### 2.1. Reagents

Fetal Bovine Serum (FBS), Dulbecco's Modified Eagle Medium (DMEM), Roswell Park Memorial Institute (RPMI) 1640 Medium, streptomycin, and penicillin were from Welgene (Daegu, Republic of Korea). The oligonucleotide primers used, shown in Table 1, were purchased from Bioneer (Daejeon, Republic of Korea). The TRIzol reagent was purchased from Invitrogen (Carlsbad, CA, USA). 2′, 7′–dichlorofluorescin diacetate (DCFDA), lipopolysaccharide (LPS), Bay-11, N-acetyl-cysteine (NAC), phorbol 12-myristate 13-acetate (PMA), and DAPI stain were obtained from Sigma–Aldrich (St. Louis, MO, USA). Antibodies for western blot analysis, inducible NO synthase (iNOS; ^#^2982), cyclooxygenase-2 (COX-2; ^#^4842), p-TAK1 (phospho-transforming growth factor beta-activated kinase 1; ^#^4531), *p*-I*κ*B*α* (^#^2859), *p*-NF*κ*B p65 (^#^3033), T-NF*κ*B p65 (^#^8242), *β*-Actin (^#^4967), total-extracellular-signal-regulated kinase (T-ERK; ^#^9102), *p*-ERK (^#^9101), total-c-Jun N-terminal kinase (T-JNK; ^#^9252), *p*-JNK (^#^9251), T-p38 (^#^9212), *p*-p38 (^#^9211), poly (ADP ribose) polymerase (PARP; ^#^9542), HRP-linked antibody (^#^7074), and ALexa Fluor 555® conjugated secondary antibody (^#^4413) were from Cell Signaling Technology (Danvers, MA, USA).

### 2.2. Preparation of DIE

The whole plant of *Duchesnea indica* was crushed, weighed, and boiled with 70% ethanol at 80°C for 2 h, concentrated via rotary evaporation, frozen, and lyophilized. The obtained powder was weighed and used in subsequent experiments.

### 2.3. Cell Culture

RAW 264.7 murine macrophage cells (TIB-71™) were from the American Type Cell Culture (ATCC) (Rockville, MD, USA) and maintained in a water-jacketed incubator supplied with 5% CO_2_ at 37°C.

### 2.4. UPLC-QToF-MS Analysis of DIE

A UPLC system equipped with a binary solvent delivery system was sourced from Waters Corp. (Milford, MA, USA), coupled with an auto-sampler and a UV detector as previously reported [22]. The quadrupole time-of-flight mass spectrometer (Q-Tof Premier™, Waters Corp., Milford, MA, USA) was used. The lock mass used was a reference solution of leucine-enkephalin ([M − H] − *m/z* 554.2615) in the form of a spray.

### 2.5. GCMS Analysis of DIE

GCMS analysis of DIE was carried out using an Agilent 7890A GC (Agilent Technologies, Santa Clara, CA, USA) as previously reported [22]. Mass spectrometry was acquired via electron ionization and scan modes.

### 2.6. Nitric Oxide (NO) and Cell Viability Assays in RAW 264.7 Cells Treated with DIE

RAW 264.7 cells were cultured at a density of 3 × 10^5^ cells/mL in 24-well plates overnight. DIE was added at 12.5 *μ*g/mL to 100 *μ*g/mL for 30 min followed by the administration of 0.1 *μ*g/mL LPS and incubated for 18 h before the supernatant was collected for NO assay using the Griess Reagent. 3-(4,5-dimethylthiazol-2-yl)-2, 5-diphenyltetrazolium bromide (MTT) was added to each well and left to incubate for 3 h. Formazan crystals of MTT were dissolved with DMSO and quantified using a plate reader (VersaMax, Molecular Devices, San Jose, CA, USA). The wavelengths used for measuring NO and MTT were 540 nm and 560 nm, respectively. Lactate dehydrogenase (LDH) activity was detected in RAW 264.7 cells with an LDH assay kit (Cayman Chemicals, Ann Arbor, MI, United States). Briefly, DIE was treated with RAW 264.7 cells as stated above, with or without LPS. LDH activity was detected according to the manufacturer's instructions.

### 2.7. RNA Extraction in RAW 264.7 Cells, Reverse Transcription Polymerase Chain Reaction (RT-PCR), and Real-Time Polymerase Chain Reaction (qRT-PCR)

RAW 264.7 cells were cultured in 6-well plates at a density of 5 × 10^5^ cells/mL and treated similarly as stated above. After 18 h, RNA extraction was done as previously described [23]. Briefly, cells were harvested using the TRIzol solution. RNA was reverse transcribed according to the manufacturer's instructions (Bioneer, Daejeon, Republic of Korea). PCR and real-time PCR were carried out as previously described [22]. Gene expression (Ct) values were normalized relative to the housekeeping gene, GAPDH. Sequences of primers used in this study are shown in Table 1.

### 2.8. Western Blot Analysis of RAW 264.7 Cells and Murine Lung Tissue

RAW 264.7 cells were cultured in 6-well plates as described above. Protein was extracted using a PRO-PREP solution (iNtRON, Daejeon, Republic of Korea). For *in vivo* studies, lungs of mice were snap frozen directly after harvesting. Cytosolic and nuclear fractions were separated using NE-PER nuclear and cytosolic reagents (Thermo Fisher Scientific, Waltham, MA, USA). Proteins were separated on 10% SDS–PAGE gels, transferred to PVDF membranes (Milipore, Burlington, MA, USA), blocked with 5% skim milk, and incubated with target antibodies (1 : 1,000) overnight at 4°C. Secondary antibody was incubated at a 1 : 3,000 dilution the following day before developing (General Electrics, Boston, MA, USA). Experiments were conducted three times and blots were quantified using ImageJ software (NIH, USA).

### 2.9. LPS-Induced ROS Damage in Murine BMDM

Bone marrow cells were harvested from the femurs of male C57/Bl6 mice, cultured in RPMI media supplemented with 15% L929 conditioned media, and allowed to differentiate for 7 days into BMDM before treatment with DIE for 30 min followed by 0.1 *μ*g/mL LPS. Cells were then incubated for an additional 18 h, stained with DCFDA, and analyzed using flow cytometry to detect LPS-induced ROS damage. NAC (10 *μ*M) was used as a reference drug.

### 2.10. Transient Transfection and Luciferase Assay of RAW 264.7 Cells

RAW 264.7 cells were transfected with NF*κ*B or AP-1 plasmid and TK-Renilla using Solfect™ (Biosolyx, Daegu, Republic of Korea) according to the manufacturer's instructions. Cells were then plated in 24-well plates at a density of 3 × 10^5^ cells/mL. After 24 h, the cells were treated with the DIE extract followed by 0.1 *μ*g/mL LPS 30 min later. Cells were incubated for 5 h and the expression of NF*κ*B and AP-1 was measured using the Promega firefly luciferase assay (Madison, WI, USA) according to the manufacturer's instructions. Luciferase activity was normalized to that of TK-Renilla luciferase.

### 2.11. Confocal Microscopy of *p*-NF*κ*B in RAW 264.7 Cells

RAW 264.7 cells were seeded onto poly-L-lysine-coated coverslips and treated with DIE and LPS as described above. After 18 h of LPS treatment, cells were fixed, washed, and incubated with 0.1% Triton-X followed by incubation with ALexa Fluor™ 555-conjugated secondary antibody and DAPI. Cells were then analyzed using Carl Zeiss LSM700 confocal laser scanning microscope (Carl Zeiss Microscopy Ltd, Cambourne, CAM, UK). The intensity of ALexa Fluor™ 555 was quantified using Image *J* (NIH, USA) and the corrected cell total fluorescence was calculated.

### 2.12. LPS-Induced Septic Shock Model in BALB/C Mice

Six-week-old male BALB/*c* mice weighing between 20 and 22 g were purchased from Orient Bio (Gyeonggi-do, Republic of Korea). Animals were left to acclimatize for one week in a pathogen-free animal facility maintained at a temperature of 22°C ± 2°C and a relative humidity of 50% ± 10%, accommodated with a 12/12 h light/dark cycle. Food and water were supplied *ad libitum*. All animal experiments were conducted and approved by the Institutional Animal Care Committee of Kyungpook National University (KNU-2019-0040). Mice were randomly separated into 5 groups, with 10 mice for survival studies and 6 mice per group for the chronic model (control, LPS, DIE 100 mg/kg, DIE 300 mg/kg, and dexamethasone 10 mg/kg). DIE and dexamethasone were administered orally for 7 days. To induce septic shock, mice were given an intraperitoneal injection of 40 mg/kg of LPS the next day and observed for a period of 5 days. For a chronic model of sepsis, mice were given an intraperitoneal injection of 15 mg/kg of LPS the following day. After 24 h of LPS administration, mice were anesthetized using 1% isoflurane. Blood, liver, lungs, and testis were harvested for further analysis.

### 2.13. TNF-*α* and NO Levels in the Serum as Measured by ELISA

Serum was separated from the blood collected via cardiac puncture and stored at −70°C until analysis. ELISA kits (R&D Systems, Minneapolis, MN, USA) were used according to the manufacturer's instructions.

### 2.14. Hematoxylin and Eosin (H&E) Staining

Harvested lungs, liver, and testis were directly fixed in 10% neutral buffered formalin. Tissues were dehydrated and fixed in paraffin before sectioning into 6-*μ*m sections and stained with H&E according to a previously reported method [24]. Histological images were acquired using Aperio ImageScope x64 software (Leica Biosystems, Buffalo Grove, IL, USA).

### 2.15. Statistical Analysis

Statistical analyses were done using GraphPad Prism version 7.00 software (San Diego, CA, USA) and one-way ANOVA was performed with Dunnett's posttest. Data are presented as mean ± SD.

## 3. Results

### 3.1. Identification of Compounds in DIE

**Figure 1:**
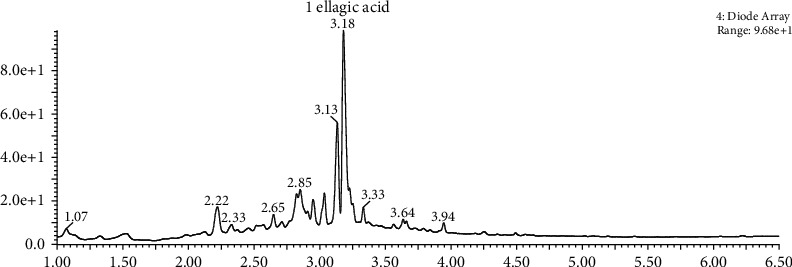
UPLC-QT of MS analysis of DIE. DIE was analyzed by UPLC-QToF-MS to identify the components. Peaks were compared to a standard of ellagic acid.

UPLC-QToF-MS analysis revealed that the main component of DIE is ellagic acid as shown in peak 1 (Figure 1). GCMS analysis revealed that the major compounds detected were 2, 3-dihydro-3, 5-dihydroxy-6-methyl-(4H)-pyran-4-one, hexadecanoic acid, 9, 12, 15-octadecatrienoic acid, gamma-sitosterol, octadecanoic acid, and leoelaidic acid (Table 2).

### 3.2. NO Assay and Cell Viability Assay in RAW 264.7 Cells

**Figure 2:**
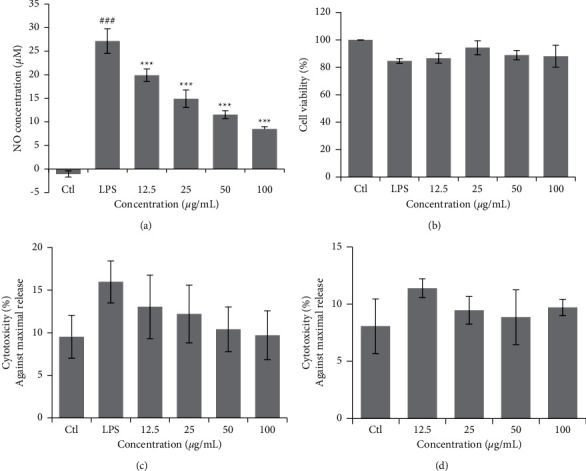
DIE suppresses LPS-induced NO production without cytotoxicity in RAW 264.7 cells. RAW 264.7 cells were seeded into 24-well plates and incubated for 24 h before treatment with DIE and LPS. Supernatants were collected after 18 h for NO analysis using the Griess reagent (a). MTT was added to the cells for 3 h and the crystals were dissolved with DMSO (b). Lactate dehydrogenase (LDH) activity was detected in RAW 264.7 cells with an LDH assay kit with LPS (c) or without LPS (d). Experiments were repeated in triplicates. Data were presented as the mean ± SD. ^#^ was *p* < 0.05, ^##^ indicates *p* < 0.01, and ^###^ indicates *p* < 0.001 as compared with the control group, whereas ^*∗*^ was *p* < 0.05, ^*∗∗*^ indicates *p* < 0.01, and ^*∗∗∗*^ indicates *p* < 0.001 as compared with the LPS-treated group.

Treatment of RAW 264.7 cells with DIE at 12.5 *μ*g/mL to 100 *μ*g/mL suppressed LPS-induced secretion of NO in a dose-dependent manner. A concentration of 100 *μ*g/mL suppressed 60% of NO induction by LPS (Figure 2(a)) and was not cytotoxic to the RAW 264.7 cells (Figure 2(b)). Cytotoxicity of DIE was also investigated using the LDH assay kit. Based on our results, there was no increased LDH release after treatment with DIE with LPS (Figure 2(c)) or without LPS (Figure 2(d)). This shows that DIE does not exhibit any cytotoxicity in RAW 264.7 cells.

### 3.3. DIE Suppresses mRNA Expression of Proinflammatory Mediators and Cytokines in RAW 264.7 Cells

**Figure 3:**
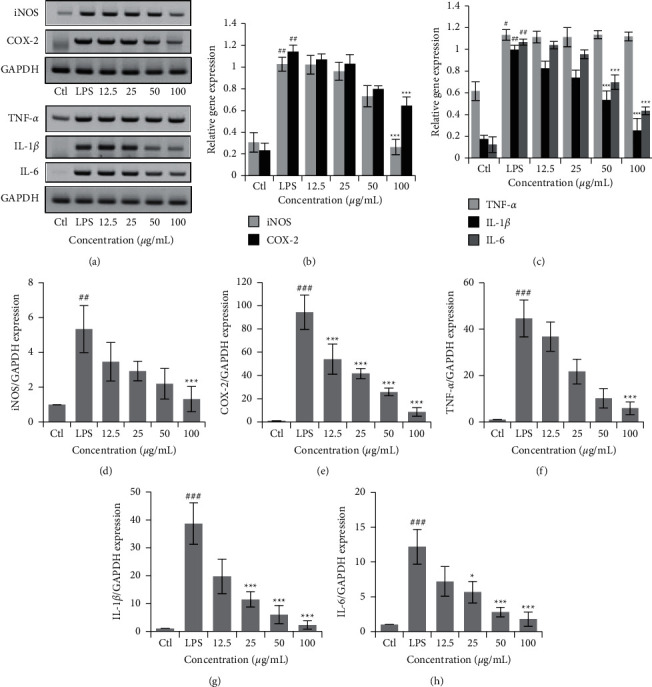
Proinflammatory mediators and cytokines in RAW 264.7 cells were suppressed by DIE. RNA was isolated from RAW 264.7 cells using TRIzoL solution. RNA was separated by choloform extraction followed by purification with alcohol. The concentration of RNA was quantified using nanophotometer and reverse transcription was carried out to synthesize cDNA. PCR was done using the cDNA with gene-specific primers. The PCR products were run on ethidium bromide-stained agarose gels and viewed with a gel developer (a). PCR was carried out in triplicate and gels were quantified using ImageJ software (b, c). Relative real-time PCR expression was normalized to the housekeeping gene, GAPDH (d–h). Full gel images were included in the additional information. Experiments were repeated in triplicates. Data are presented as mean ± SD. ^#^ was *p* < 0.05, ^##^ indicates *p* < 0.01, and ^###^ indicates *p* < 0.001 as compared with the control group, whereas ^*∗*^ was *p* < 0.05, ^*∗∗*^ indicates *p* < 0.01, and ^*∗∗∗*^ indicates *p* < 0.001 as compared with the LPS-treated group.

DIE effectively suppressed iNOS, COX-2, IL-1*β*, and IL-6 as investigated by RT-PCR (Figure 3(a)) and quantified using ImageJ software (NIH, USA) in Figures 3(b) and 3(c). Real-time PCR revealed that all proinflammatory mediators and cytokines were significantly suppressed by DIE (Figures 3(d)–3(h)).

### 3.4. DIE Suppresses the Expression of Proteins of the NF*κ*B and MAPK Signaling Pathways

**Figure 4:**
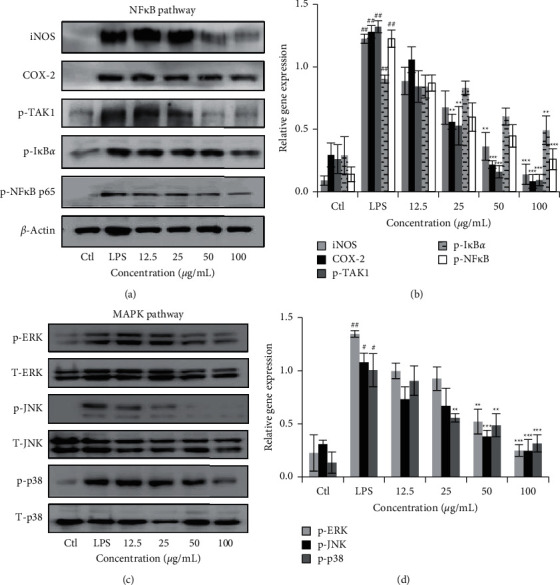
DIE suppresses protein expression in the NF*κ*B and MAPK pathways in RAW 264.7 cells. Proteins that were extracted from RAW 264.7 cells were separated using 10% SDS–PAGE followed by a transfer onto PVDF membranes. Membranes were blocked with skim milk and incubated with corresponding primary antibodies overnight. Membranes were then incubated with secondary antibody and developed. Representative gel images for the NF*κ*B and MAPK pathways are shown (a, c). Western blot analysis was done in triplicate and quantified using ImageJ software. Proteins in the NF*κ*B pathway were quantified and normalized against the housekeeping gene, *β*-actin (b), whereas proteins in the MAPK pathway were normalized against their total forms (d). Full blot images were included in the additional information. Experiments were repeated in triplicate. Data are presented as mean ± SD. ^#^ was *p* < 0.05, ^##^ indicates *p* < 0.01, and ^###^ indicates *p* < 0.001 as compared with the control group, whereas ^*∗*^ was *p* < 0.05, ^*∗∗*^ indicates *p* < 0.01, and ^*∗∗∗*^ indicates *p* < 0.001 as compared with the LPS-treated group.

LPS is a ligand of TLR4, which activates the NF*κ*B and MAPK pathways. Our results indicated that increasing the doses of DIE suppressed the expression of phospho-TAK1, phospho-NF*κ*B p65, *p*-I*κ*B*α*, iNOS, COX-2, and proteins in the MAPK pathway (Figures 4(a) and 4(c)). Gel images were quantified using ImageJ software (NIH, USA) (Figures 4(b) and 4(d)). The expression of the NF*κ*B pathway proteins was measured relative to the housekeeping gene, *β*-Actin, and phosphorylated proteins in the MAPK pathway were normalized against their total forms.

### 3.5. DIE Reduces LPS-Induced Oxidative Stress in Murine BMDM and Suppresses the Expression of NF*κ*B in RAW 264.7 Cells

**Figure 5:**
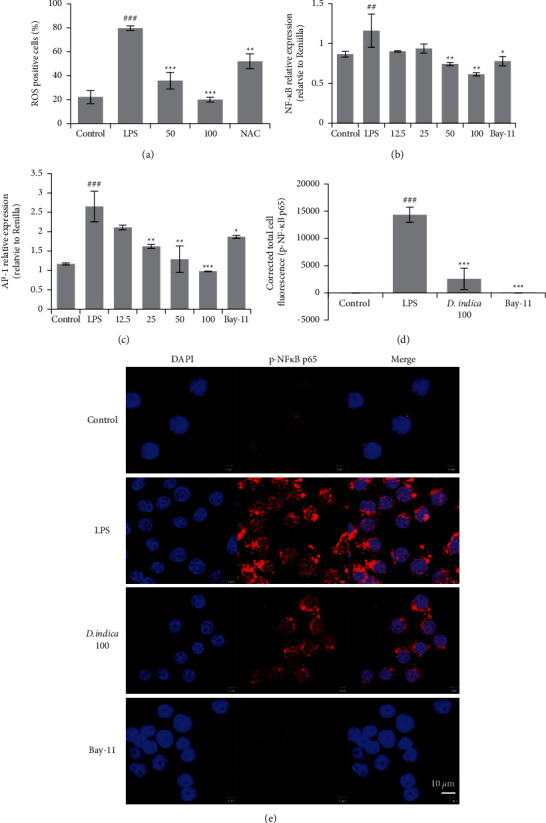
DIE attenuates oxidative damage and prevents the translocation of phospho-NF*κ*B in RAW 264.7 cells. Bone marrow cells were isolated and differentiated into BMDM. LPS was administered to induce oxidative stress and ROS was measured using DCFDA. Flow cytometry was carried out for quantification (a). pNF*κ*B-Luc and pAP-1-Luc were transfected into RAW 264.7 cells. Cells that were treated with LPS were analyzed for the expression of phospho-NF*κ*B and AP-1 by luciferase assay (b, c). Confocal microscopy was used to investigate the translocation of phospho-NF*κ*B p65 in RAW 264.7 cells. Fluorescence quantification was carried out with ImageJ software and the corrected total cell fluorescence was calculated (d). Images of confocal microscopy where the scale bar represents 10 *μ*m (e). Data are presented as mean ± SD. ^#^ was *p* < 0.05, ^##^ indicates *p* < 0.01, and ^###^ indicates *p* < 0.001 as compared with the control group, whereas ^*∗*^ was *p* < 0.05, ^*∗∗*^ indicates *p* < 0.01, and ^*∗∗∗*^ indicates *p* < 0.001 as compared with the model group.

The analysis of ROS by flow cytometry revealed that treatment with 50 and 100 *μ*g/mL DIE significantly reduced DCFDA-positive cells (Figure 5(a)). Using luciferase assay following treatment with DIE, NF*κ*B expression was decreased significantly but AP-1 was increased (Figures 5(b) and 5(c)). Bay-11 was used as a reference drug. RAW 264.7 cells that were treated with or without LPS and DIE were subjected to confocal microscopy to observe the translocation of NF*κ*B p65 into the nucleus. We observed an increase in the expression of phospho-NF*κ*B p65 in cells treated with LPS. A merged image showed the expression of phospho-NF*κ*B p65 in the nucleus, indicating the translocation of phospho-NF*κ*B p65. Following treatment with DIE, the expression of phospho-NF*κ*B p65 appeared to be weaker and its expression was lower in the nucleus (Figure 5(e)). The intensity of *p*-NF*κ*B p65 was quantified with ImageJ and the corrected total cell fluorescence indicated that DIE reduced *p*-NF*κ*B p65 significantly (Figure 5(d)).

### 3.6. DIE Increases Mortality in an Acute Model of LPS-Induced Septic Shock and Improves Markers in a Chronic Model of Sepsis

**Figure 6:**
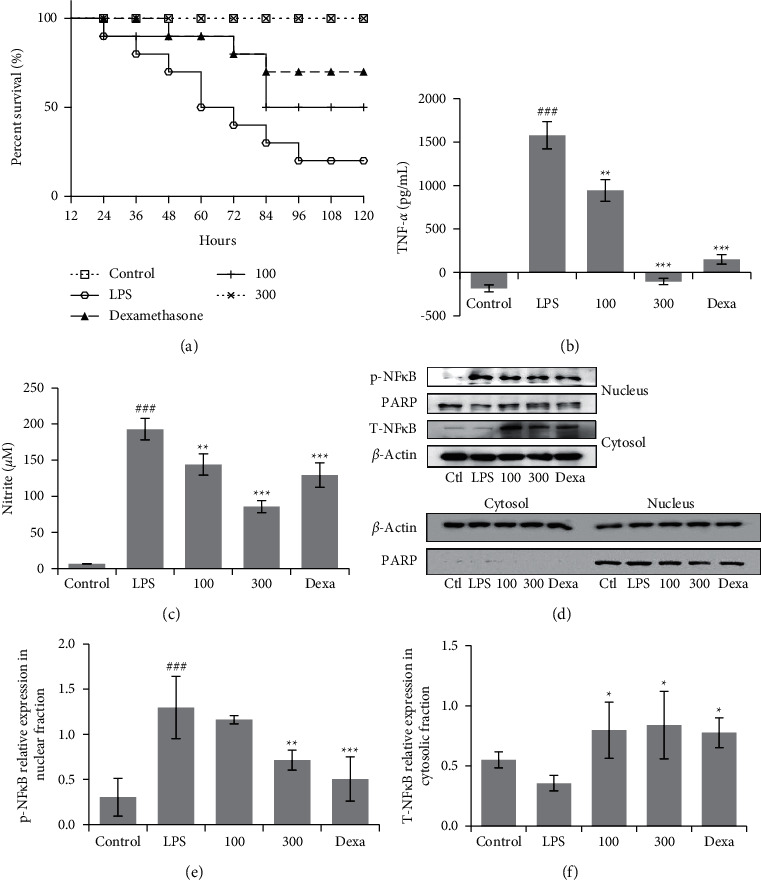
DIE rescues mice induced with septic shock and attenuates inflammatory markers in mice. Mice treated with DIE and dexamethasone (Dexa) were administered a single dose of LPS *i.p.* after 7 days of oral treatment. The mortality of the mice was monitored (a) (*n* = 10 per group). Serum TNF-*α* levels and nitrite concentration were determined using an ELISA assay (b, c). Lung tissues were harvested after euthanization. The expression of phospho-NF*κ*B in the nucleus and total NF*κ*B in the cytosol of lung tissue were investigated by western blot analysis. The cytosolic fraction was detected for PARP to ensure purity of the separated fractions (d). Blots were quantified using ImageJ software and the expression in the nucleus was normalized against PARP and the expression in the cytosol was normalized against *β*-Actin (e) and (f). Full blot images were included in the additional information. Data are presented as mean ± SD. ^#^ indicates *p* < 0.05, ^##^ indicates *p* < 0.01, and ^###^ indicates *p* < 0.001 as compared with the control group, whereas ^*∗*^ was *p* < 0.05, ^*∗∗*^ indicates *p* < 0.01, and ^*∗∗∗*^ indicates *p* < 0.001 as compared with the LPS-treated group.

Administration of DIE to mice reduced their mortality induced with septic shock in a dose-dependent manner (Figure 6(a)). In a separate model of sepsis, serum levels of TNF-*α* and nitrite were significantly decreased in a dose-dependent manner with increasing doses of DIE (Figures 6(b) and 6(c)). The expression of phospho-NF*κ*B was suppressed in the nucleus with DIE treatment, whereas the total NF*κ*B expression recovered in the cytosol in murine lung tissue. The expression of PARP was also not detected in the cytosolic fraction (Figure 6(d)). Quantification of blots, carried out using ImageJ software, indicated that DIE treatment induced a significant decrease in phospho-NF*κ*B while significantly increasing total NF*κ*B (Figures 6(e) and 6(f)).

### 3.7. DIE Improves Histopathological Damage Induced by LPS

**Figure 7:**
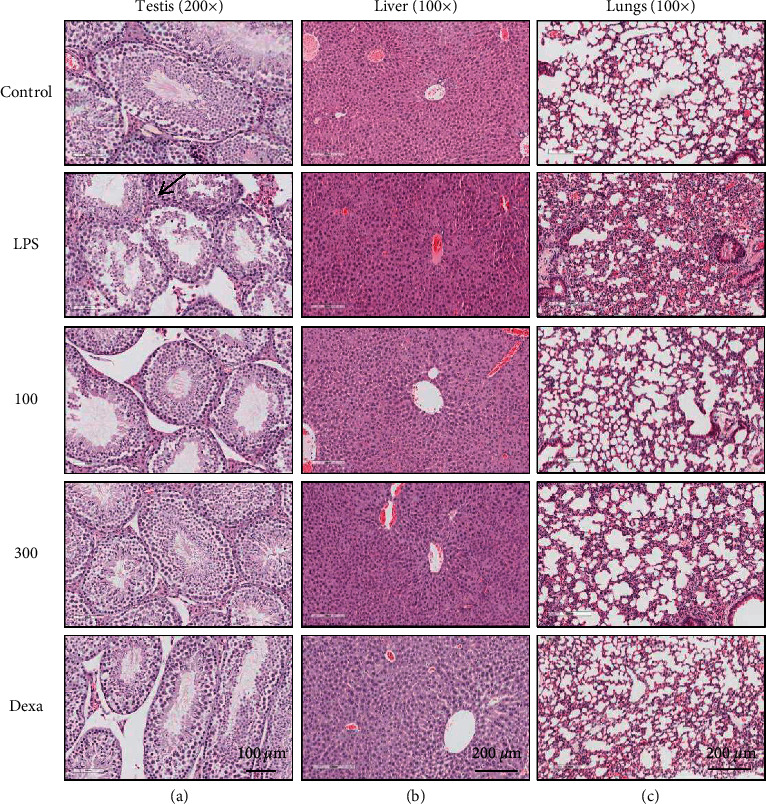
DIE prevents histopathological damage in mice induced in a chronic model of sepsis. After euthanization, the testis (a), liver (b), and lungs (c) were directly fixed in 10% neutral buffered formalin and stained with H&E. The respective magnification of the testis was 200x and 100x for the lungs and the liver. The scale bar for the testis group indicates 100 *μ*m, whereas the scale bars for the liver and lungs indicate 200 *μ*m. The arrow indicates the destruction of the seminiferous tubular margin and diminished sperm flagella in the testis. Dexamethasone was indicated as Dexa.

LPS induced the destruction of seminiferous tubule structure (as indicated by arrow) and the lack of sperm flagella in the testis of mice, which has been reversed by DIE treatment to a level similar to that of the reference drug, dexamethasone (Figure 7(a)). In the liver of LPS-induced mice, accumulation or red blood cells were visible, indicating backflow congestion of draining pathways. This was also restored by DIE treatment (Figure 7(b)). Lungs in LPS-induced mice had destroyed alveolar structure accompanied with increased infiltration of neutrophils. Treatment with DIE prevented the destruction of the alveolar structure and reduced neutrophil infiltration, indicating a reduced inflammatory response (Figure 7(c)).

## 4. Discussion

In sepsis, neutrophils are activated and it secretes toxic products that damage nearby cells. In septic patients, the lifespan of neutrophils is extended due to delayed neutrophil apoptosis caused by the prolonged activation of NF*κ*B and suppressed caspace 9 levels [25]. The NF*κ*B pathway is well-known for its role in the inflammatory response and is associated with the activation of NF*κ*B pathway with sepsis [26–28]. In addition, reports show the relevance of the MAPK pathway with sepsis [24, 29, 30]. Therefore, we targeted the activation of the NF*κ*B and MAPK pathways in this study. Macrophages respond to inflammation by secreting proinflammatory cytokines like TNF-*α*, IL-1*β*, and IL-6. Their secretion activates the inflammatory cascade, increases NO secretion, and consequently induces damage to the surrounding tissues. Our *in vitro* studies confirmed that DIE is a potential anti-inflammatory agent. DIE has the ability to reduce LPS-induced NO secretion, suppress mRNA expression of proinflammatory cytokines in RAW 264.7 cells, and prevent ROS damage in murine BMDM. ROS are the product of many cellular processes and can be upregulated with increased cytokine and growth factor receptor activity. NF*κ*B regulates ROS levels, and vice versa; ROS can also regulate the expression of NF*κ*B through various cellular processes [31]. Our study shows that DIE is a potent anti-inflammatory agent that targets ROS to inhibit the NF*κ*B activation. This potential was also reported in Panax ginseng, which potently targets oxidative stress and the NF*κ*B [32, 33].

Although a previous study has already reported that the whole plant extract of *Duchesnea indica* exhibits anti-inflammatory effects in RAW 264.7 cells [14], its efficacy in reversing sepsis has yet to be studied. DIE manages to rescue mice with septic shock and prevents the translocation of NF*κ*B into the lungs of mice with sepsis. As sepsis is known to cause organ failure, many reports have been focused on sepsis-induced acute lung injury [34–36]. Therefore, we investigated the expression of NF*κ*B in the lungs of mice. DIE has effectively prevented the translocation of *p*-NF*κ*B into the nucleus in the lungs of mice in a model of sepsis. We also observed the histopathological damage caused by LPS in the lungs, testis, and liver of mice. Our findings revealed that DIE has effectively prevented LPS-induced impairment in them.

UPLC-QToF-MS analysis revealed that one of the main compounds in DIE is ellagic acid. Ellagic acid is a polyphenol that can be sourced from various sources [37–41] and has been reported to exert a variety of beneficial effects including anti-pigmentation [42], antioxidant effects [43], anti-inflammatory, and anti-coagulatory activities [44]. Furthermore, ellagic acid has also been reported to reverse liver damage in rats by suppressing the NF*κ*B signaling pathway [45]. This supports our findings and suggests that ellagic acid may be the bioactive compound responsible for rescuing mice from induced septic shock. We have also conducted GCMS analysis to analyze non-polar compounds in DIE. 4H-Pyran-4-one, 2, 3-dihydro-3, 5-dihydroxy-6-methyl-4(H)-pyran-4-one was detected and reports have shown that it effectively suppresses the proliferation of human colon cancer cells by inactivating NF*κ*B [46]. Hexadecanoic acid, commonly known as palmitic acid, was also identified in DIE and has reported to be an anti-inflammatory compound [47]. Another major molecule detected in DIE was 9, 12, 15-octadecatrienoic acid, commonly known as alpha-linoleic acid. It has been known to prevent inflammatory and lipid cardiovascular risk in hypercholesterolemic patients [48]. These results support the conclusion that DIE efficiently rescues mice from induced septic shock by potently inhibiting NF*κ*B signaling. Sepsis may induce a flared or suppressed immune response which varies among patients. Although DIE has been reported for its immune regulatory properties and anti-inflammatory properties in murine alveolar macrophages [20, 49], further studies should be conducted to investigate whether DIE has immune stimulatory properties in a separate model of sepsis-induced immunosuppression.

In conclusion, our study showed that DIE is a potential therapeutic agent for the treatment of sepsis. DIE effectively rescued mice induced with septic shock. It also reduced the levels of inflammatory markers in the serum of mice in a chronic model of sepsis by suppressing the translocation of NF*κ*B into the nucleus of lung cells. Potential drugs can take a long time to develop. Natural alternatives like DIE are widely consumed due to their relatively lower toxicity. As sepsis involves a series of inflammatory cascades, DIE can also be used as a supplementation to existing drugs used to treat sepsis due to its potent anti-inflammatory properties. Future studies can also be conducted to validate the efficacy of DIE in humans as a potential agent to treat sepsis.

## Figures and Tables

**Table 1:** Primer sequences used in this study.

RT-PCR	
GAPDH	F: 5′-CACTCACGGCAAATTCAACGGCAC-3′ R: 5′-GACTCCACGACATACTCAGCAC-3′
iNOS	F: 5′-CCCTTCCGAAGTTTCTGGCAGCAGC-3′ R: 5′-GGCTGTCAGAGCCTCGTGGCTTTGG-3′
COX-2	F: 5′-CACTACATCCTGACCCACTT-3′ R: 5′-ATGCTCCTGCTTGAGTATGT-3′
TNF-*α*	F: 5′-TTGACCTCAGCGCTGAGTTG-3′ R: 5′-CCTGTAGCCCACGTCGTAGC-3′
IL-1*β*	F: 5′-CTGTGGAGAAGCTGTGGCAG-3′ R: 5′-GGGATCCACACTCTCCAGCT-3′
IL-6	F: 5′-GTACTCCAGAAGACCAGAGG-3′ R: 5′-TGCTGGTGACAACCACGGCC-3′

Real-time PCR	
GAPDH	F: 5′-CACTCACGGCAAATTCAACGGCAC-3′ R: 5′-GACTCCACGACATACTCAGCAC-3′
iNOS	F: 5′-GGCAGCCTGTGAGACCTTTG-3′ R: 5′-GCATTGGAAGTGAAGCGTTTC-3′
COX-2	F: 5′-GGCAGCCTGTGAGACCTTTG-3′ R: 5′-GCATTGGAAGTGAAGCGTTTC-3′
TNF-*α*	F: 5′-TGCCTATGTCTCAGCCTCTTC-3′ R: 5′-GAGGCCATTTGGGAACTTCT-3′
IL-1*β*	F: 5′-CAACCAACAAGTGATATTCTCCATG-3′ R: 5′-GATCCACACTCTCCAGCTGCA-3′
IL-6	F: 5′-TCCAGTTGCCTTCTTGGGAC-3′ R: 5′-GTGTAATTAAGCCTCCGACTTG-3′

**Table 2:** Compounds identified in DIE by GCMS analysis.

Area%	Compound
20.37	2, 3-Dihydro-3, 5-dihydroxy-6-methyl-(4H)-pyran-4-one
18.04	Hexadeconic acid
11.1	9, 12, 15-Octadecatrienoic acid
7.25	Gamma-sitosterol
5.52	Octadecanoic acid
4.54	Leoelaidic acid

## Data Availability

All data produced during the study are included in this manuscript.

## References

[1] Singer M., Deutschman C. S., Seymour C. W. (2016). The third international consensus definitions for sepsis and septic shock (Sepsis-3). *JAMA*.

[2] Lumley J. W., Siu S. K., Rllay S. P. (1992). Single dose ceftriaxone as prophylaxis for sepsis in colorectal surgery. *ANZ Journal of Surgery*.

[3] Eykyn S. J., Phillips I. (1976). Metronidazole and anaerobic sepsis. *British Medical Journal*.

[4] Zhao Z., Wang B., Mu L. (2020). Long-term exposure to ceftriaxone sodium induces alteration of gut microbiota accompanied by abnormal behaviors in mice. *Frontiers in Cellular and Infection Microbiology*.

[5] Wang L., Li S., Liu H., Bao L. (2020). Advances in research on the effects of natural drugs with immune-promoting effects on immune function. *European Journal of Inflammation*.

[6] Hotchkiss R. S., Moldawer L. L., Opal S. M., Reinhart K., Turnbull I. R., Vincent J.-L. (2016). Sepsis and septic shock. *Nature Reviews Disease Primers*.

[7] Chow J. C., Young D. W., Golenbock D. T., Christ W. J., Gusovsky F. (1999). Toll-like receptor-4 mediates lipopolysaccharide-induced signal transduction. *Journal of Biological Chemistry*.

[8] Lee J. H., Min D. S., Lee C. W., Song K. H., Kim Y. S., Kim H. P. (2018). Ginsenosides from Korean Red Ginseng ameliorate lung inflammatory responses: inhibition of the MAPKs/NF-*κ*B/c-Fos pathways. *Journal of Ginseng Research*.

[9] Gai Z., Visentin M., Gui T. (2018). Effects of farnesoid X receptor activation on arachidonic acid metabolism, NF-*κ*B signaling, and hepatic inflammation, and hepatic inflammation. *Molecular Pharmacology*.

[10] Chen P. N., Yang S. F., Yu C. C. (2017). Duchesnea indica extract suppresses the migration of human lung adenocarcinoma cells by inhibiting epithelial–mesenchymal transition. *Environmental Toxicology*.

[11] Yang W.-E., Ho Y.-C., Tang C.-M. (2019). Duchesnea indica extract attenuates oral cancer cells metastatic potential through the inhibition of the matrix metalloproteinase-2 activity by down-regulating the MEK/ERK pathway. *Phytomedicine*.

[12] Xiang B., Yu X., Li B., Xiong Y., Long M., He Q. (2019). Characterization, antioxidant, and anticancer activities of a neutral polysaccharide from *Duchesnea indica* (Andr.) Focke. *Journal of Food Biochemistry*.

[13] Long M., Yu X., Li B., Xiong Y., Xiang B., He Q. (2020). Characterisation of antioxidant, anti-inflammatory, and immunomodulatory activities of polysaccharides derived from Duchesnea indica (Andrews) Focke. *International Food Research Journal*.

[14] Zhao L., Zhang S.-L., Tao J.-Y. (2008). Anti-inflammatory mechanism of a folk herbal medicine,*Duchesnea indica* (andr) focke at RAW264.7 cell line. *Immunological Investigations*.

[15] Zhu M., Dong X., Guo M. (2015). Phenolic profiling of Duchesnea indica combining macroporous resin chromatography (MRC) with HPLC-ESI-MS/MS and ESI-IT-MS. *Molecules*.

[16] Miao Q. (2010). Study on chemical constituents of duchesnea indica Andr. Focke. *Academic Journal of Second Military Medical University*.

[17] Pietta P.-G. (2000). Flavonoids as antioxidants. *Journal of Natural Products*.

[18] Sharma N., Biswas S., Al-Dayan N., Alhegaili A. S., Sarwat M. (2021). Antioxidant role of kaempferol in prevention of hepatocellular carcinoma. *Antioxidants*.

[19] Mittal M., Siddiqui M. R., Tran K., Reddy S. P., Malik A. B. (2014). Reactive oxygen species in inflammation and tissue injury. *Antioxidants and Redox Signaling*.

[20] Tafani M., Sansone L., Limana F. (2016). The interplay of reactive oxygen species, hypoxia, inflammation, and sirtuins in cancer initiation and progression. *Oxidative Medicine and Cellular Longevity*.

[21] Kumar S., Gupta E., Kaushik S., Kumar Srivastava V., Mehta S., Jyoti A. (2018). Evaluation of oxidative stress and antioxidant status: correlation with the severity of sepsis. *Scandinavian Journal of Immunology*.

[22] Lee Y. Y., Kim M., Irfan M. (2020). Ulmus parvifolia jacq. exhibits antiobesity properties and potentially induces browning of white adipose tissue. *Evidence-Based Complementary And Alternative Medicine*.

[23] Saba E., Irfan M., Jeong D. (2019). Mediation of antiinflammatory effects of Rg3-enriched red ginseng extract from Korean red ginseng via retinoid X receptor *α*–peroxisome-proliferating receptor *γ* nuclear receptors. *Journal of ginseng research*.

[24] O’Sullivan A. W., Wang J. H., Redmond H. P. (2009). NF-*κ*B and P38 MAPK inhibition improve survival in endotoxin shock and in a cecal ligation and puncture model of sepsis in combination with antibiotic therapy. *Journal of Surgical Research*.

[25] Taneja R., Parodo J., Jia S. H., Kapus A., Rotstein O. D., Marshall J. C. (2004). Delayed neutrophil apoptosis in sepsis is associated with maintenance of mitochondrial transmembrane potential and reduced caspase-9 activity. *Critical Care Medicine*.

[26] Luo C., Liu Z., Xu Y. (2019). Protective effects of mesenchymal stem cells on acute liver injury via TLR4/NF-*κ*B signaling pathway in sepsis mice. *International Journal of Clinical and Experimental Medicine*.

[27] Selvaraj V., Nepal N., Rogers S. (2015). Inhibition of MAP kinase/NF-*κ*B mediated signaling and attenuation of lipopolysaccharide induced severe sepsis by cerium oxide nanoparticles. *Biomaterials*.

[28] Feketeova E., Li Z., Joseph B., Shah R., Spolarics Z., Ulloa L. (2018). Dopaminergic control of inflammation and glycemia in sepsis and diabetes. *Frontiers In Immunology*.

[29] Song G. Y., Chung C.-S., Jarrar D., Cioffi W. G., Ayala A. (2002). Mechanism of immune dysfunction in sepsis: inducible nitric oxide-meditated alterations in p38 MAPK activation. *The Journal of Trauma, Injury, Infection, and Critical Care*.

[30] Guo W., Liu W., Chen G. (2012). Water-soluble andrographolide sulfonate exerts anti-sepsis action in mice through down-regulating p38 MAPK, STAT3 and NF-*κ*B pathways. *International Immunopharmacology*.

[31] Bubici C., Papa S., Pham C., Zazzeroni F., Franzoso G. (2006). The NF-kB-Mediated Control of ROS and JNK Signaling. *Histology And Histopathology*.

[32] Lee S. Y., Kim M. H., Kim S. H. (2021). Korean red ginseng affects ovalbumin-induced asthma by modulating IL-12, IL-4, and IL-6 levels and the NF-kB/COX-2 and PGE2 pathways. *PGE2 pathways, Journal of Ginseng Research*.

[33] Huang W. C., Huang T. H., Yeh K. W., Chen Y. L., Shen S. C., Liou C. J. (2021). Ginsenoside Rg3 ameliorates allergic airway inflammation and oxidative stress in mice. *Journal of Ginseng Research*.

[34] Filgueiras L. R., Martins J. O., Serezani C. H., Capelozzi V. L., Montes M. B. A., Jancar S. (2012). Sepsis-induced acute lung injury (ALI) is milder in diabetic rats and correlates with impaired NFkB activation. *PLoS One*.

[35] Wang J., Liu Y.-T., Xiao L., Zhu L., Wang Q., Yan T. (2014). Anti-inflammatory effects of apigenin in lipopolysaccharide-induced inflammatory in acute lung injury by suppressing COX-2 and NF-*κ*B pathway. *Inflammation*.

[36] Yang L., Li D., Zhuo Y., Zhang S., Wang X., Gao H. (2016). Protective role of liriodendrin in sepsis-induced acute lung injury. *Inflammation*.

[37] Mullen W., Yokota T., Lean M. E., Crozier A. (2003). Analysis of ellagitannins and conjugates of ellagic acid and quercetin in raspberry fruits by LC–MSn. *Phytochemistry*.

[38] da Silva Pinto M., Lajolo F. M., Genovese M. I. (2008). Bioactive compounds and quantification of total ellagic acid in strawberries (Fragaria x ananassa Duch. *Food Chemistry*.

[39] Vattem D. A., Shetty K. (2003). Ellagic acid production and phenolic antioxidant activity in cranberry pomace (Vaccinium macrocarpon) mediated by Lentinus edodes using a solid-state system. *Process Biochemistry*.

[40] Papoutsi Z., Kassi E., Chinou I., Halabalaki M., Skaltsounis L. A., Moutsatsou P. (2008). Walnut extract (Juglans regia L.) and its component ellagic acid exhibit anti-inflammatory activity in human aorta endothelial cells and osteoblastic activity in the cell line KS483. *British Journal of Nutrition*.

[41] Panichayupakarananta P., Issuriya A., Sirikatitham A., Wang W. (2010). Antioxidant assay-guided purification and LC determination of ellagic acid in pomegranate peel. *Journal of Chromatographic Science*.

[42] Yoshimura M., Watanabe Y., Kasai K., Yamakoshi J., Koga T. (2005). Inhibitory effect of an ellagic acid-rich pomegranate extract on tyrosinase activity and ultraviolet-induced pigmentation. *Bioscience Biotechnology and Biochemistry*.

[43] Festa F., Aglitti T., Duranti G., Ricordy R., Perticone P., Cozzi R. (2001). Strong antioxidant activity of ellagic acid in mammalian cells in vitro revealed by the comet assay. *Anticancer Research*.

[44] Chao P.-C., Hsu C.-C., Yin M.-C. (2009). Anti-inflammatory and anti-coagulatory activities of caffeic acid and ellagic acid in cardiac tissue of diabetic mice. *Nutrition and Metabolism*.

[45] Aslan A., Gok O., Erman O., Kuloglu T. (2018). Ellagic acid impedes carbontetrachloride-induced liver damage in rats through suppression of NF-*κ*B, Bcl-2 and regulating Nrf-2 and caspase pathway. *Biomedicine and Pharmacotherapy*.

[46] Ban J. O., Hwang I. G., Kim T. M. (2007). Anti-proliferate and pro-apoptotic effects of 2,3-dihydro-3,5-dihydroxy-6-methyl-4H-pyranone through inactivation of NF-*κ*B in human colon cancer cells. *Archives of Pharmacal Research*.

[47] Aparna V., Dileep K. V., Mandal P. K., Karthe P., Sadasivan C., Haridas M. (2012). Anti-inflammatory property of n-hexadecanoic acid: structural evidence and kinetic assessment. *Chemical Biology and Drug Design*.

[48] Zhao G., Etherton T. D., Martin K. R., West S. G., Gillies P. J., Kris-Etherton P. M. (2004). Dietary *α*-linolenic acid reduces inflammatory and lipid cardiovascular risk factors in hypercholesterolemic men and women. *Journal of Nutrition*.

[49] Ullah H. M. A., Lee Y. Y., Kim S. D., Rhee M. H. (2021). Duchesnea indica extract attenuates coal fly ash-induced inflammation in murine alveolar macrophages through the NF-kappa B pathway. *Evidence-Based Complementary and Alternative Medicine*.

